# Combining dual-tree complex wavelets and multiresolution in iterative CT reconstruction with application to metal artifact reduction

**DOI:** 10.1186/s12938-019-0727-1

**Published:** 2019-12-05

**Authors:** Defne Us, Ulla Ruotsalainen, Sampsa Pursiainen

**Affiliations:** 1 Laboratory of Signal Processing, Tampere University, Korkeakoulunkatu 1, 33720 Tampere, Finland; 2 Laboratory of Mathematics, Tampere University, Korkeakoulunkatu 3, 33720 Tampere, Finland

**Keywords:** Cone beam computed tomography (CT), Dual-tree complex wavelet transform, Iterative reconstruction, Metal artifact reduction, Multiresolution

## Abstract

**Background:**

This paper investigates the benefits of data filtering via complex dual wavelet transform for metal artifact reduction (MAR). The advantage of using complex dual wavelet basis for MAR was studied on simulated dental computed tomography (CT) data for its efficiency in terms of noise suppression and removal of secondary artifacts. Dual-tree complex wavelet transform (DT-CWT) was selected due to its enhanced directional analysis of image details compared to the ordinary wavelet transform. DT-CWT was used for multiresolution decomposition within a modified total variation (TV) regularized inversion algorithm.

**Methods:**

In this study, we have tested the multiresolution TV (MRTV) approach with DT-CWT on a 2D polychromatic jaw phantom model with Gaussian and Poisson noise. High noise and sparse measurement settings were used to assess the performance of DT-CWT. The results were compared to the outcome of the single-resolution reconstruction and filtered back-projection (FBP) techniques as well as reconstructions with Haar wavelet basis.

**Results:**

The results indicate that filtering of wavelet coefficients with DT-CWT effectively removes the noise without introducing new artifacts after inpainting. Furthermore, adoption of multiple resolution levels yield to a more robust algorithm compared to varying the regularization strength.

**Conclusions:**

The multiresolution reconstruction with DT-CWT is also more robust when reconstructing the data with sparse projections compared to the single-resolution approach and Haar wavelets.

## Background

Cone beam computed tomography (CBCT) has been increasingly used over the past decade as it provides information on bone size, presence of a wide variety of materials, surrounding anatomical structures such as nerves and sinuses, precise localization of implant placement sites, and surgical planning decisions [1, 2]. With the increased acceptance, affordability and accessibility of metallic restorations in forms of dental implants, fillings, crowns, screws, nails, prosthesis and plates in dentistry, and the increasing popularity of CBCT in image-guided therapy, dental CT specific metal artifact reduction (MAR) algorithms became a field of its own in the scientific research [3]. The attenuation of high density objects such as stainless steel, gold alloys, silver amalgam, platinum, lead, tin and aluminum, can corrupt the images of the underlying anatomical structures in dental CT, allowing fewer photons to reach detectors. This photon starvation corrupts the projection data, leading to streak artifacts over the surrounding tissue upon back-projection. These artifacts can reduce the applicability of dental CT by hindering the underlying anatomical structures [4]. For recent applications of MAR in the field of CT ranging from its use in positron emission tomography scans to spinal deformity correction in surgeries, see [5, 6]. The latest comparison of the available MAR algorithms from the largest vendors have also been tested with a customized phantom by Chou et al. [7]. For the effectiveness of MAR with various metals in CT, readers can refer to [8].

The aim of MAR methods is to remove artifacts caused by the presence of metallic objects in the reconstructed images. MAR methods can be generally divided into two main categories: (1) interpolation/completion of projection data and (2) iterative reconstruction methods. The former approach is not sufficient in complicated cases such as multiple metals [9]. The combination of these two categories is also possible and it can further improve the reconstruction results. An overview of these methods is provided in [10].

Inpainting is one of the most commonly used projection completion methods due to its high computational efficiency [9]. It is an interpolation based method for filling the missing information in an image by interpolating the information surrounding it. Inpainting was introduced in signal processing by [11] and it has been widely used in MAR in projection domain [9, 12] and wavelet domain [13]. In practice, inpainting replaces the gaps in the data with NaNs and then fill them by interpolating the intensity values surrounding the NaNs. The inpainting methods in this work was implemented via the code of John D’Errico [14].^1^ As the following multiresolution reconstruction method already is an iterative method, inpainting was chosen here instead of iterative approaches to optimize the efficiency of the algorithm. Although inpainting fills the gaps in an image efficiently, it can lead to secondary artifacts during analytic reconstruction due to discontinuities at the boundary pixels, e.g., at the metal-tissue boundary. In order to prevent such artifacts, we propose filtering the projection data in dual complex wavelet basis within a multiresolution framework, which combines inpainting [14] with iterative total variation (TV) reconstruction. This combination is motivated as complementary with respect to correcting the primary and secondary effects of the metals, that is, the missing data intensity profile and details, respectively. The multiresolution iterative total variation (MRTV) is an extension of the classical single-resolution TV iteration [15–17]. It utilizes a coarse-to-fine approach, in which the coarse image details are reconstructed before the finer ones to enhance the regularity, suppress the noise, and avoid the secondary artifacts after inpainting [18–20]. Namely, under missing data, only coarse level details might be distinguishable and methods not taking this into account might have a poor performance or numerical instability with respect to these details.

The multiresolution decomposition in MRTV have been successfully applied in MAR to resolve some of such issues related to the existing methods [4, 12, 20]. In [20], a wavelet-based filtering for MAR was applied with CT data acquired for a hip joint prosthesis, and it was found to be effective in reducing the artifacts from beam hardening and photon starvation. Following a similar reasoning, we chose to use wavelet coefficients to distinguish different frequency components and filter the high-frequency artifacts caused by metals and noise without disturbing the edges of the object. For achieving the best possible performance, we applied the dual-tree complex wavelet transform (DT-CWT) [21–23]. The DT-CWT is based on two real discrete wavelet transforms (DWTs), which give the real and imaginary parts of the DT-CWT separately. As a directionally accurate transform, 2D DT-CWT can recognize the orientation of the image fluctuations, making it considerably less sensitive to the artifacts related to alteration or compression of the coefficients as compared to the classical wavelets, e.g., Daubechies or biorthogonal wavelets used in [20]. The complex wavelet transform (CWT) achieves perfect reconstruction and the dual-tree approach ensures this when decomposition level is greater than one [24]. In contrast to the ordinary 2D wavelet transform, which includes vertical, horizontal and diagonal direction modes, DT-CWT oversamples the target image with a doubled directional selectivity. Consequently, it distinguishes both ascending and descending curves in the image, whereas DWT does not. This is essential for preserving the reconstruction quality as good as possible. The advantages of DT-CWT was utilized within the multiresolution framework in order to achieve good noise filtering without filtering out the details in the image. In this study, our goal is to find out, how MRTV approach performs compared to the ordinary single-resolution TV (SRTV) regularization and also to the classical filtered back-projection (FBP) technique, which is used as the reference method to evaluate the performances of other methods presented here.

In the numerical experiments, the MRTV approach was found to stabilize the reconstructions compared to SRTV. Differences between the methods investigated were observed, especially, in regions of interest (ROIs) containing metals and their near surroundings. The influence of angular density on the reconstructions was studied by using different number of projections. The results with sparse projections would be relevant with respect to lowering the total radiation dose [25, 26]. Additionally, the stability of the algorithm against the total number of projections could make it applicable for various CBCTs available on the market. For instance, in 2013, the number of projections acquired ranged from 180 to 1024. The Kodak CS 9300C CBCT device utilizes 180 projections for a total rotation angle of 180 degrees, while most devices deliver 360 projections per full angle rotation [27].

## Results

**Fig. 1:**
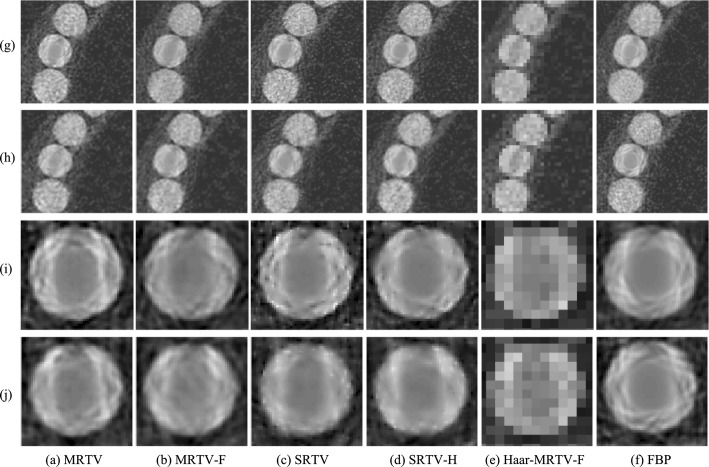
Reconstruction results for Configurations I (noisy) and II (noisy and sparse). Rows labeled with (g) and (h) depict the parts of the reconstructed images near ROI 2 for Configurations I and II, respectively. Rows (i) and (j) present the images from ROI 3 for Configurations I and II, respectively. All images covering the same region are shown within the same color range

The resulting images from the reconstructions are presented in Fig. 1. The secondary artifacts in FBP around ROI 2 are slightly less pronounced with the DT-CWT filtering step. These artifacts are almost completely vanished once multiresolution approach is combined with DT-CWT. The images reconstructed with Haar wavelets are so pixelized that it is not possible to evaluate the secondary artifacts. When images with the tooth within ROI 3 are visually assessed, the same observations for the ROI 2 still apply. Additionally, in SRTV, artifacts caused by single-resolution filtering are visible, but these artifacts are decreased by the increased penalty weight in SRTV-H. The contrast difference between the tooth and the inpainted metal is pronounced in the single resolution images and the FBP, whereas this difference is significantly less with MRTV and MRTV-H.

The quantitative evaluation of the results, using RMSE, PSNR and SSIM, is depicted in Table 1. For Configurations I (noisy) and II (noisy and sparse), the multiresolution approach with DT-CWT fared better compared to single-resolution approaches. In general, the filtering of wavelet coefficients in MRTV-F improved the RMSE and PSNR values for Configuration II. In Configuration I, however, the filtering deteriorated the PSNR and RMSE despite the marginal improvement in SSIM. Increasing the penalty weight in SRTV improved all quantitative parameters for Configurations I and II. Due to the pixelization in the reconstruction with Haar wavelets, its RMSE was higher than other methods even in the noiseless measurements. In the case of Configuration III (noiseless data), all the methods with DT-CWT yield to similar results due to the preliminary stage optimization of the reconstruction parameters. For dense projection data in Configuration I, the multiresolution with wavelets (both Haar and DT-CWT) performed better than single-resolution approaches in ROI 1. For the sparse projections in Configuration II, MRTV with DT-CWT outperformed the Haar wavelets.

**Table 1:** The quantitative evaluation of the reconstructions computed in the numerical experiments

Type	Conf.	Region	MRTV	MRTV-F	SRTV	SRTV-H	Haar-MRTV-F	FBP
RMSE	I	Full image	0.324	0.336	0.356	0.346	0.325	0.316
	I	ROI 1	0.211	0.212	0.225	0.220	0.210	0.207
	I	ROI 2	0.066	0.074	0.070	0.071	0.072	0.066
	I	ROI 3	0.059	0.067	0.061	0.062	0.066	0.061
	II	Full image	0.318	0.310	0.341	0.319	0.332	0.355
	II	ROI 1	0.206	0.202	0.215	0.207	0.215	0.223
	II	ROI 2	0.068	0.066	0.072	0.066	0.071	0.072
	II	ROI 3	0.061	0.061	0.064	0.061	0.064	0.065
	III	Full image	0.256	0.258	0.253	0.254	0.294	0.258
	III	ROI 1	0.176	0.176	0.177	0.177	0.194	0.176
	III	ROI 2	0.059	0.058	0.058	0.058	0.069	0.059
	III	ROI 3	0.054	0.054	0.053	0.053	0.062	0.054
	I	Full image	9.79	9.47	8.97	9.21	9.77	10.01
	I	ROI 1	13.50	13.47	12.97	13.15	13.56	13.69
	I	ROI 2	23.62	22.61	23.10	22.94	22.82	23.67
	I	ROI 3	24.56	23.47	24.35	24.18	23.59	24.38
	II	Full image	9.94	10.17	9.35	9.94	9.59	8.99
	II	ROI 1	13.71	13.90	13.34	13.67	13.35	13.03
	II	ROI 2	23.39	23.59	22.86	23.54	23.02	22.84
	II	ROI 3	24.29	24.34	23.88	24.35	23.83	23.75
	III	Full image	11.85	11.77	11.94	11.90	10.64	11.78
	III	ROI 1	15.08	15.07	15.03	15.05	14.26	15.07
	III	ROI 2	24.67	24.73	24.80	24.71	23.51	24.59
	III	ROI 3	25.43	25.41	25.53	25.47	24.08	25.41
SSIM	I	Full image	0.16	0.24	0.10	0.13	0.31	0.16
	I	ROI 1	0.834	0.839	0.829	0.831	0.841	0.836
	I	ROI 2	0.993	0.993	0.993	0.993	0.993	0.993
	I	ROI 3	0.996	0.996	0.996	0.996	0.995	0.996
	II	Full image	0.21	0.28	0.14	0.17	0.21	0.10
	II	ROI 1	0.839	0.843	0.835	0.838	0.834	0.829
	II	ROI 2	0.993	0.993	0.993	0.993	0.992	0.993
	II	ROI 3	0.996	0.996	0.996	0.996	0.995	0.995
	III	Full image	0.72	0.71	0.76	0.77	0.55	0.67
	III	ROI 1	0.910	0.905	0.915	0.917	0.858	0.901
	III	ROI 2	0.994	0.994	0.995	0.995	0.993	0.994
	III	ROI 3	0.996	0.996	0.996	0.996	0.995	0.996

Note that SSIM values are not directly comparable between different subimages or ROIs, since SSIM is not invariant with respect to image size

**Fig. 2:**
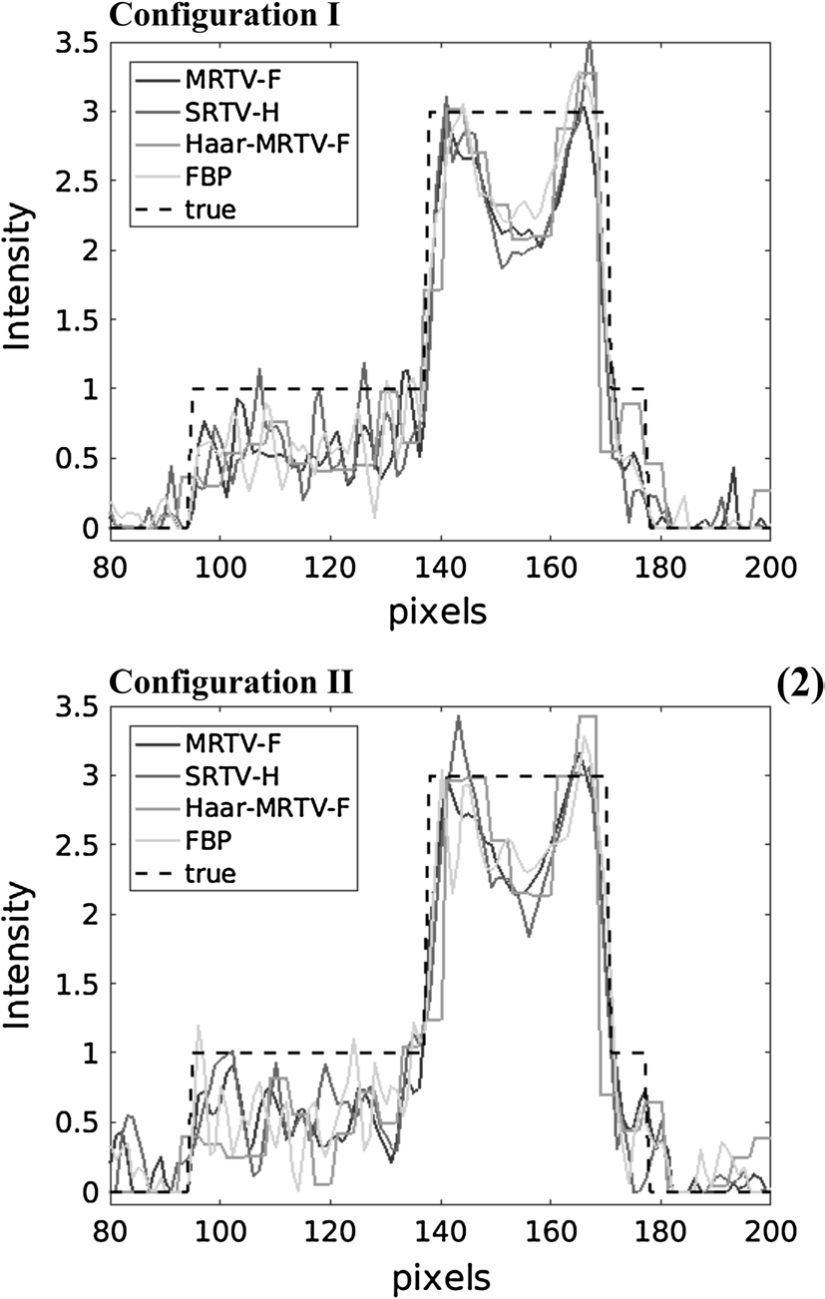
Horizontal line profiles for Configurations I and II. Only the line profiles of MRTV-F, SRTV-H, Haar-MRTV-F, and FBP are depicted here for clarity of the figure. The line profiles have been calculated over the red line in Fig 3a

**Fig. 3:**
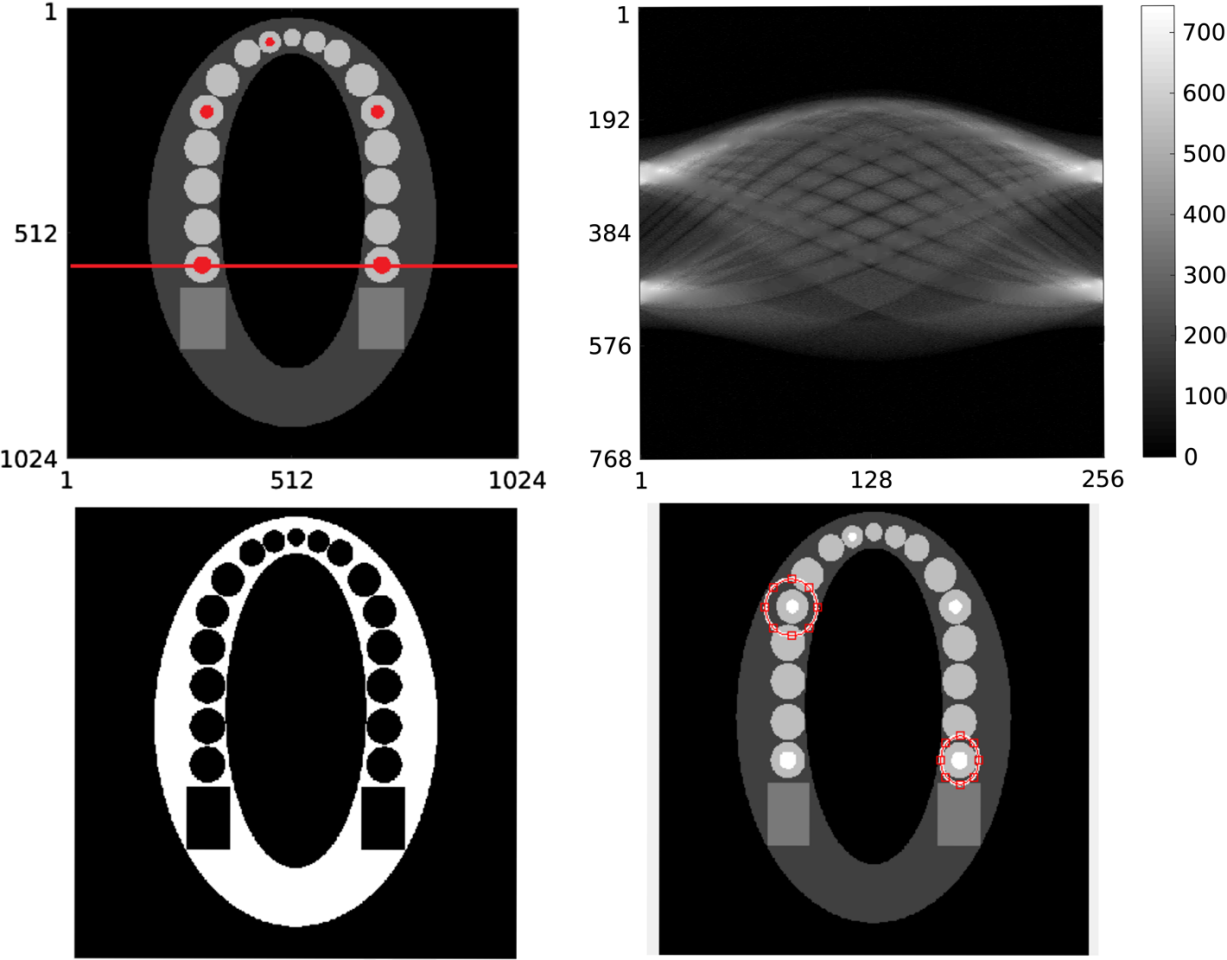
The dataset and ROIs. **a** The metallic regions are marked red on the phantom. **b** The resolution of the phantom, from which the sinogram is calculated, is $1024 \times 1024$ pixels. The noisy projection data after inpainting has the resolution of $768 \times 256$ pixels. **c** Region of interest (ROI) 1 consisting of the soft tissue (white) surrounding the teeth. **d** ROI 2 and ROI 3 correspond to the encircled areas. Each of them includes a single tooth with metallic implant

The line profiles in Fig. 2 were calculated along the red line in Fig. 3. Based on these line profiles, it can be seen that the MRTV with wavelet filtering suppresses the noise better than SRTV with a high penalty (SRTV-H). The pixelization of the Haar wavelet reconstruction is also visible in the line profile. The fluctuations of SRTV-H and Haar-MRTV-F near the metallic region become more apparent in Configuration II, while MRTV profile is closer to the ground truth.

The CPU time for the MRTV and SRTV reconstruction process, implemented in single computing thread, was 725 and 232 s, respectively. The FBP was obtained in 0.15 s.

## Discussion

This study focused on enhancing the reconstruction quality of iterative regularization via the dual-tree complex wavelet transform (DT-CWT) [21–23] in dental CT, combined with multiresolution. Although FBP resulted in comparable values of RMSE and SSIM with complete data and low noise scenarios, the difference of the proposed method became apparent with sparse data. The central finding of this study was that the DT-CWT equipped MRTV inversion technique was more robust in terms of reduction of noise and artifacts for sparse data. This observation was supported by the numeric evaluations and visual comparisons. Although part of this robustness of the reconstruction compared to FBP can be attributed to TV penalization, the difference in error and similarity measures of Haar and DT-CWT point at the importance in selection of the coefficients to be filtered.

Based on our results, DT-CWT provided virtually an artifact-free multiresolution basis, which can be observed based on the nearly identical outcome of MRTV and SRTV in the case of the noiseless data (Configuration III). The conventional wavelets used in the preliminary tests, in particular, the Haar basis [28], led to pixelization of the final reconstruction. That is, the correction steps for the finer resolutions did not match accurately enough with the coarse level estimate. Hence, DT-CWT was found to be vital for the appropriate function of MRTV. Some ringing effects were observed for the individual resolution levels, but, the final estimate did not suffer from ringing. Other potential multiresolution bases for MRTV are provided by ridgelets and curvelets [29–31] which similarly to DT-CWT cover an extended set of orientations compared to the classical wavelets.

Sinogram denoising with a 80 % hard threshold (MRTV-F) improved the RMSE values with sparse projections (Configuration II). However, the RMSE results of the dense projections with filtering were inferior to the outcome obtained with MRTV despite the improvement in SSIM, suggesting that some details were lost in the thresholding process along with some noise reduction. This suggests that additional denoising in single resolution is a not as effective technique recovery of the intensity values as employing a multiresolution decomposition in iterative reconstruction. We emphasize that present hard threshold filter in MRTV-F can be improved, e.g., via a soft threshold and regional adaptivity, especially, regarding the metal implants.

Using multiple resolution levels was also found to be preferable compared to controlling the regularization strength. With sparse projection data used in Configuration II, the SRTV-H performed equally well compared to MRTV in terms of RMSE, possibly due to the strong penalization of the noise. With SRTV-H, the overall image quality could be improved with respect to the artifacts by increasing the level of the regularization, but, with the cost of decreased image sharpness. The line profiles, however, showed a high positive bias for the tooth around the metal and lower intensity values for the metallic implant. In contrast, MRTV achieved an enhanced accuracy for the coarse details while maintaining the sharpness at the level of SRTV. Another important observation was that MRTV successfully reconstructed both 256 and 128 projection angles utilized in Configuration I and II, respectively. In general, the coarse-to-fine reconstruction approach seems to be advantageous regarding MAR, where reconstructing the implanted teeth accurately can be difficult due to the inpainted sinogram regions and, thereby, the incompleteness of the data. As suggested by the present study, recovering the coarse level fluctuations before the finer ones can result in a more accurate tooth boundaries than, if the whole image is reconstructed at once. This can be understood, since for the present inverse problem the numerical null space $S_{\varepsilon }^-$ [19, 32] is non-trivial and there is infinitely many candidate solutions which fit the incomplete data. Hence, in addition to TV, a multiresolution setting akin to the present one might work also with other reconstruction approaches. Note that it is possible to change the multiresolution levels depending on the spatial resolution of the image. For instance, for a $256 \times 256$ image, the resolution level would be 3, while 5 levels could be chosen for a $1024 \times 1024$ image.

An important direction for future work is to validate the present DT-CWT based MRTV approach in 3-dimensional clinical dental CT data. For that purpose, the current implementation of MRTV needs to be sped up. The matrix-based MRTV implementation of this study utilized only a single computing thread and was, thereby, far from optimal with respect to a multi-thread CPU performance. Consequently, it required several minutes of CPU time, whereas the FBP reconstruction could be obtained in a fraction of a second. A parallelized matrix-free implementation would obviously accelerate the MRTV. Another potential solution would be to employ a graphics processing unit (GPU) for the inverse computations instead of a CPU, which might enable a 10–100 times faster performance based on the general performance difference between GPUs and CPUs. An analogous computationally intensive future direction would be to finding optimized ways to grow the imaging resolution *per se* without remarkably extending the computing time. The denoising technique used in MRTV-F can also be improved in order to achieve optimal imaging results. In addition to the sinogram, also the reconstruction can be filtered using DT-CWT. This approach was omitted in this study, as it did not enhance the RMSE compared to MRTV in the preliminary tests. To fully understand the effects of the noise, for example, with respect to the instrument-specific factors, such as the interplay between the detector response and the beam hardening effects, it will be essential to use real experimental or clinical measurement data in the future studies.

## Conclusion

In this work, we showed how DT-CWT can be applied in the tomographic reconstruction process via a multiresolution (coarse-to-fine) version of a classical TV regularization algorithm. The numerical experiments were aimed at minimizing the reconstruction errors due to the inpainting of metallic regions in the projection data. The multiresolution technique (MRTV) was compared to the single-resolution TV approach, for which a lower and higher regularization strength (SRTV and SRTV-H) was used. The results were also compared with reconstructions using Haar wavelet basis. Qualitative and quantitative results showed that data filtering with DT-CWT combined with multiresolution reconstruction is beneficial for recovering the details of images while reducing the noise with filtering at each resolution level. The robustness of the reconstruction with sparse projections using DT-CWT indicates the feasibility of these wavelets especially for sparse measurements. This could potentially help decreasing the radiation dose by reconstructing high quality images from sparse projection angles.

## Materials and methods

### Dataset preparation

As the simulation dataset (Table 2), we used the density map (unit g/cm$^3$) of a two-dimensional 1024 $\times$ 1024 pixel jaw phantom. This dataset was based on the FORBILD jaw phantom.^2^ Metal (golden crown), teeth, jaw bone (cortical), soft tissue (modeled as water) and air gap inside the mouth were modeled with density values of 19.32, 2.99 (enamel), 1.92, 1.00 and 0 g/cm$^3$, respectively. The locations for metallic implants in the image and projection domains can be seen in Fig. 3 as well as regions of interest (ROIs). In order to avoid committing “inverse crime” during the reconstruction, the sinogram was constructed on a fine grid of 1024 pixels, then reconstructed on a 512-pixel grid, similar to the approach of Nuyts et al. [33]. The projection data consisted of 768 radial bins and 256 angular views, covering 180 degrees. For a reference, industrial datasets might have a resolution of 600-pixels [25].

**Table 2:** The essential dataset parameters

Parameter	Specification	Value
Resolution (pixels)	Phantom	1024 $\times$ 1024
	Sinogram	768 × 512
Density (g/cm^3^)	Metal	19.32
	Teeth	2.99
	Jaw bone	1.92
	Soft tissue	1.00
	Air	0
Peak kilovoltage (kVp)	X-ray beam	80
Photon count per pixel	Emission (Poisson noise)	$10^{5}$
Standard deviation	Gaussian noise	10
Number of projections	Configuration I	256
	Configuration II	128
	Configuration III	256

For modeling of the beam hardening, a polychromatic beam model was used. The beam hardening in this context refers to the “hardening” of the beam as it passes through the object being scanned, meaning that the lower energy rays are attenuated more than the higher energy ones. The hardening of the beam at the detector end is not modeled, as the algorithms of the manufacturers often account and correct for this effect already on the raw projection data. The energy dependent mass attenuation coefficients (with coherent scattering) of gold, bone, hard tissue and soft tissue were obtained from the National Institute of Standards and Technology (NIST) database.^3^ The mass attenuation coefficient for the tooth was approximated using the material composition of enamel from [34] and NIST database.^4^ The 80 kVp spectrum (half-value layer (Al) of about 5.5 mm) was used with 1 mm Al filtration from Fessler’s IRT toolbox [35]. As the cone beam itself creates additional artifacts due to the shape of the beam, the parallel beam approach was selected for the construction of the system matrix. This allows one to evaluate the effectiveness of the MAR methods specifically on the artifacts created by the metals without the influence of the cone beam. The possible geometrical artifacts due to parallel beams were omitted here as the emphasis was on the effect of noise. Both Poisson and Gaussian noise were modeled in the sinogram construction, following the description of [36], which was also used in TIGRE Toolbox.^5^ For Poisson noise, the total emitted photon count per pixel ($I_{0}$) was taken as $10{^5}$ and a zero mean additive Gaussian noise was used with standard deviation of 10. In order to maintain the generality of the model, the instrument-specific details such as the detector response were omitted in this study.

Three different measurement settings were used to evaluate the algorithm’s performance against noise and sparsity of measurements. In the first one (Configuration I), the number of projections was 256 with Poisson and Gaussian noise. In Configuration II, the noise model was the same, while a sparse pattern of 128 projections was applied to investigate the effects of the projection count which in some of the clinical scanners is fewer than in I [27]. In Configuration III, the projection pattern of I was used without the Gaussian noise to assess the performance of the single and multiresolution methods under more ideal conditions without changing the count statistics.

The metals were extracted by global thresholding from the projection data. For the sake of simplicity in evaluating the performance of the suggested methods, perfect segmentation of the metals was assumed. The gaps left on the sinogram after metal extraction were filled via inpainting.

### Methodology

#### Dual-tree complex wavelet transform

The ordinary real (orthogonal) DWT [28, 37] is based on a low- and high-pass filter function $\phi : {\mathbb {R}} \rightarrow {\mathbb {R}}$ and $\psi : {\mathbb {R}} \rightarrow {\mathbb {R}}$ which together enable decomposing a given signal *f*(*t*) as given by

1 $$\begin{aligned} f(t) \! = \! \sum _{k = - \infty }^\infty \alpha _k \, \phi (t - k) + \sum _{\ell = 0}^\infty \sum _{k = - \infty }^\infty \beta _{k,\ell } \, 2^{\ell /2} \psi (2^\ell t - k), \end{aligned}$$

with $\alpha _k$ and $\beta _{k,\ell }$ denoting the so-called approximation and detail coefficients, respectively. The filter functions are orthogonal and normalized to one, i.e., the product between two different filter functions integrated over the real line is zero and ${\int _{-\infty }^\infty \phi (t - k)^2 \, \hbox {d} t } = {\int _{-\infty }^\infty 2^\ell \psi (2^\ell t - k)^2 \, \hbox {d} t } = 1$. Consequently, the coefficients $\alpha _k$ and $\beta _{k,\ell }$ can be obtained via the following integrals:

2 $$\begin{aligned} \alpha _k= & {} \int _{-\infty }^\infty f(t) \phi (t - k) \, \hbox {d} t, \end{aligned}$$

3 $$\begin{aligned} \beta _{k,\ell }= & {} \int _{-\infty }^\infty f(t) 2^{\ell /2} \psi (2^\ell t - k) \, \hbox {d} t. \end{aligned}$$

Furthermore, the DWT conserves signal energy, meaning that the Parseval’s identity holds:

4 $$\begin{aligned} \int _{-\infty }^{\infty } f(t)^2 \, \hbox {d} t = \sum _{k = - \infty }^\infty \alpha _k^2 + \sum _{\ell = 0}^\infty \sum _{k = - \infty }^\infty \beta _{k,\ell }^2. \end{aligned}$$

Together the coefficients can be organized into a tree-structured hierarchy of multiple resolution levels: each level has two branches, one for low- and one for high-pass filter coefficients.

The two-dimensional filter functions can be obtained as separable products between their one-dimensional counterparts, i.e., $\phi (x,y) = \phi (x) \phi (y)$, $\psi _H(x,y) = \phi (x) \psi (y)$, $\psi _V(x,y) = \psi (x) \phi (y)$, and $\psi _D(x,y) = \psi (x) \psi (y)$. The high-pass filters $\psi _H(x,y)$, $\psi _V(x,y)$, and $\psi _D(x,y)$ correspond to a horizontal, vertical and diagonal directional mode, respectively. Characteristic to the 2D DWT is that, due to their symmetry in the Fourier domain, these modes do not distinguish between upward and downward slopes in the image [23]. Consequently, DWT easily produces checkerboard-like dense and non-directional artifacts around edges, if the coefficients are altered or compressed. The lowest-order case of the DWT is constituted by the piecewise constant Haar wavelets which have been previously used together with TV in reconstruction [13, 38]. Therefore, it was also used here for comparison.

In DT-CWT, the low- and high-pass filter function is assumed to be of the form

5 $$\begin{aligned} \phi (t) = \phi _h(t) + j \phi _g(t) \quad \hbox {and} \quad \psi (t) = \psi _h(t) + j \psi _g(t), \end{aligned}$$

where $\phi _h(t), \phi _g(t), \psi _h(t)$, and $\psi _g(t)$ are real functions. The dual-tree structure follows as each of the pairs $\phi _h(t), \psi _h(t)$ and $\phi _g(t), \psi _g(t)$ forms a real-valued and orthogonal wavelet-tree.

The two-dimensional high-pass filters of the DT-CWT have altogether six directional modes [23], corresponding to the real part of the separable products $\phi (x) \psi (y)$, $\phi (x) \overline{\psi (y)}$, $\psi (x) \phi (y)$, $\psi (x) \overline{\phi (y)}$, $\psi (x) \psi (y)$, and $\psi (x) \overline{\psi (y)}$ and the angular orientations of − 63, 63, − 27, 27, − 45, and 45 degrees with respect to the* x*-axis, respectively. Of these, the first two are nearly horizontal, 3rd and 4th one nearly vertical and the last two are diagonal.

#### Total variation regularization

The goal of any image reconstruction in a linear system is to invert the equation

6 $$\begin{aligned} \mathbf{y} = \mathbf{L} \mathbf{x} + \mathbf{n}, \end{aligned}$$

where $\mathbf{x}$ is the image to be reconstructed, the vector $\mathbf{y}$ contains the measurement (projection) data, the matrix $\mathbf{L}$ is a discretized Radon transform (Radon matrix). This system is an idealized expression for the signal attenuation and measurement process. It is introduced and used here for deriving the further mathematical equations. In fact, the entries of the Radon matrix contain some uncertainty, as the X-ray photon emission is a Poisson process, and $\mathbf{n}$ is a measurement noise term. A regularized solution of (6) can be obtained through the following:

7 $$\begin{aligned} \mathbf{x }_{\ell +1} = (\mathbf{L}^T \mathbf{L} + \mathbf{D} {\varvec{\Gamma }}_{\ell } \mathbf{D} )^{-1} \mathbf{L}^T \mathbf{y}, \end{aligned}$$

where ${\varvec{\Gamma }}_{\ell }$ is a weighting matrix that satisfies ${\varvec{\Gamma }}_0 = \mathbf{I}$ and ${\varvec{\Gamma }}_{\ell } = \hbox {diag} ( |\mathbf{D} \mathbf{x_{\ell }} | + \gamma \mathbf{I})^{-1}$ for $\ell \ge 1$ with a suitably chosen regularization parameter $\gamma \ge 0$. $\mathbf{D}$ is the regularization matrix given by

8 $$ \begin{aligned}   D_{{i,j}} &=  \frac{{\alpha (2\delta _{{i,j}}  - 1)\int_{{{\text{P}}_{i}  \cap {\text{P}}_{j} }} {\text{d}}s}}{{\max _{{i,j}} \int_{{{\text{P}}_{i}  \cap {\text{P}}_{j} }} {\text{d}}s}} + \beta \delta _{{i,j}} ,\quad {\text{with}} \hfill \\   \delta _{{i,j}}  & =  \left\{ {\begin{array}{*{20}l}    {1,} \hfill & {{\text{if }}j = i,} \hfill  \\    {0,} \hfill & {{\text{otherwise}},} \hfill  \\   \end{array} } \right. \hfill \\  \end{aligned}  $$

with $\mathrm {P}_i$ and $\mathrm {P}_j$ denoting the boundary of the $i{th}$ and $j{th}$ pixel, respectively. Their intersection coincides with the edges shared by these pixels. The governing regularization parameter $\alpha$ determines the strength of the TV regularization. The roles of $\beta$ and $\gamma$ are mainly to ensure the invertibility of the matrices $\mathbf{D}$ and ${\varvec{\Gamma }}_\ell$ so that the TV iteration does not diverge. The first term of $\mathbf{D_{i,j}}$ in (8) penalizes the jumps over the pixel edges and the second one corresponds to the norm of $\mathbf{x}$. In this work, $\beta$ was fixed at 10$^{-8}$. The conjugate gradient method was applied for matrix inversion with the number of steps fixed to 100. If this iteration converges, it minimizes the regularized objective function $F(\mathbf{x}) = \Vert \mathbf{L} \mathbf{x} - \mathbf{y} \Vert ^2_2 + 2 \Vert \mathbf{D}{} \mathbf{x} \Vert _1$ in which the l1 norm of $\mathbf Dx$ is the total variation of $\mathbf{x}$ , if $\beta = 0$ [39]. Consequently, the reconstructed image is likely to have large connected subsets close to constant, which helps to reduce noise, while preserving the edges. In this study, we call (7) the single-resolution TV (SRTV) approach. The SRTV-H refers to the stronger penalization of TV with a larger $\alpha$ value.

#### Multiresolution TV regularization

We propose approaching MAR via a multiresolution TV (MRTV) technique, that is, a coarse-to-fine extension (see Appendix) of the algorithm in (7). To explain this idea, we introduce the following definition of the numerical null-space [19, 32]:

9 $$\begin{aligned} S_\varepsilon ^- = \{ x \, | \, \Vert \mathbf{L x} \Vert \le \varepsilon \Vert \mathbf{x} \Vert \}. \end{aligned}$$

Here $\varepsilon$ denotes the floating-point accuracy, which is mainly concentrated on the fine image fluctuations. We assume that the target spaces of the wavelet low- and high-pass filter pair provide approximations of the space of strongly suppressed image details $S_\varepsilon ^-$ and that of the well-detectable details $S_\varepsilon ^+ = \{ 0 \} \cup \{ x \, |\, \Vert \mathbf{L x} \Vert > \varepsilon \Vert \mathbf{x} \Vert \}$, respectively. These spaces decompose the candidate solution space as given by ${\mathbb {R}}^n = S_\varepsilon ^+ \oplus S_\varepsilon ^-$. The aim of the coarse-to-fine approach is to separate $S_\varepsilon ^+$ and $S_\varepsilon ^-$ in the reconstruction process in order to maximize the distinguishability of the details belonging to $S_\varepsilon ^-$. Processing the coarse details before the finer ones can approximately separate the strongly suppressed fluctuations of $S_\varepsilon ^-$ from the well-detectable ones belonging to the space $S_\varepsilon ^+ = \{ 0 \} \cup \{ x \, |\, \Vert \mathbf{L x} \Vert > \varepsilon \Vert \mathbf{x} \Vert \}$. The low- and high-pass wavelet filters can be obtained via a wavelet decomposition by zeroing all the high-pass and low-pass coefficients, respectively. In other words, the reconstruction of each wavelet level helps in separating the fine image details from the undesired components of the image such as noise and artifacts.

### Numerical experiments

The present reconstruction approach was validated with numerical experiments using the jaw phantom described earlier. The reconstruction procedure included the following four stages:

1. Detecting the metals in the sinogram via global thresholding,
2. Laplacian smoothed inpainting of the metals using the algorithm in [14],
3. DT-CWT denoising with a given hard threshold percent (0% or 80%),
4. Inversion of the data via the MRTV, MRTV-F, SRTV, SRTV-H, or FBP technique.

The hard threshold refers to the percentage of the smallest wavelet coefficients which are set to zero. It aims to further reduce the noise in the sinogram before reconstruction. In MRTV-F, with 80% threshold, only the largest 20% of the wavelet coefficients were used in the reconstruction. The DT-CWT was used in the inversion stage (4) to obtain the multiresolution decomposition for MRTV.

The regularization parameter values were chosen empirically. MRTV, MRTV-F and SRTV were optimized for Configuration III. The minimal level of regularization sufficient to suppress any staircase patterns was sought for SRTV. The regularization strength applied in the case of MRTV was matched roughly with that of SRTV. In SRTV-H, slightly higher value of $\alpha$ was used for an enhanced noise tolerance. For SRTV and SRTV-H, it was necessary to choose $\gamma > 0$, and it was set to $\gamma =$ 10$^{-2}$. For MRTV, the optimal performance was obtained with $\gamma =0$. The number of MRTV and SRTV iteration steps taken in computing a single reconstruction was set to be three.

The number of nested resolution levels used in MRTV computations and denoising was set to four. The multiresolution inverse estimates computed without and with DT-CWT denoising are referred to as MRTV and MRTV-F, respectively. The regularization parameter $\alpha$ was chosen empirically as 4. MRTV results were compared with FBP and single-resolution estimates SRTV and SRTV-H, for which the corresponding $\alpha$s are 15 and 20, respectively. In FBP, the Hamming filter with a high-frequency cut-off of 1 was used in order to decrease high-frequency artifacts. Although all configurations that were implemented for DT-CWT were also implemented with Haar wavelets, the best overall performing reconstruction with Haar wavelets is depicted in the results, which was found to be filtered multiresolution approach, denoted with Haar-MRTV-F. The details for MRTV, MRTV-F, SRTV, SRTV-H, FBP and Haar-MRTV-F are included in Table 3

**Table 3:** Details for the reconstructions computed in the numerical experiments

Name	Levels	Filter	Used wavelet coefficients (%)	$\alpha$
MRTV	4	–	100	4
MRTV-F	4	DT-CWT	20	4
SRTV	1	–	100	15
SRTV-H	1	–	100	20
FBP	1	Hamming	–	–
Haar-MRTV	4	Haar	100	4

.

The results were quantitatively analyzed for 3 ROIs as well as the full image (see Fig. 3). ROI 1 corresponds to the soft tissue surrounding the teeth and ROIs 2 and 3 include a single tooth with gold implant. The denoising performance of the reconstruction methods were analyzed via the root mean squared error (RMSE) and peak signal-to-noise ratio (PSNR), in which the jaw phantom without metals was taken as the ground truth. At the locations of the metal implants, the intensity values of the ground truth vector was set to be equal to the intensity value of the teeth. Structural similarity index (SSIM) was used to evaluate the similarity of the reconstructed images to the ground truth in all ROIs [40]. The SSIM is 1 when the reference image is identical to the image to be evaluated. As the similarity between images decrease, so does the SSIM value.

All the scripts were written using MATLAB version R2016b. To run the computations, we used a high-end Lenovo P510 workstation equipped with one Intel Xeon E5-2620v4 central processing unit (CPU) and 192 GB RAM. The projection matrices for the multiresolution transform were stored as sparse arrays. The iterative MRTV and SRTV reconstruction procedures were obtained by evaluating the Radon and wavelet transforms explicitly as sparse matrices in a single computing thread. For the FBP, MATLAB’s built-in iradon function was used.

## Data Availability

Please contact with the corresponding author.

## Appendix: Multiresolution (coarse-to-fine) approach

In the present multiresolution version of the TV regularization (see 7) the coarse details are reconstructed before the finer ones. We utilize the DT-CWT in this procedure via the projection matrices ${\mathcal {P}}_\phi$ and ${\mathcal {P}}_\psi$ which multiplied with an image vector $\mathbf{x}$ yield a coefficient vector for the low- and high-pass filter at a given resolution level. Furthermore, we define a filtered Radon matrix for the coarse and fine level as $\mathbf{L}_\phi = \mathbf{L} {{\mathcal {P}}}_\phi$ and $\mathbf{L}_\psi = \mathbf{L} {{\mathcal {P}}}_\psi$. Denoting $\mathbf{G}_\phi = (\mathbf{L}_\phi ^T \mathbf{L}_\phi + \mathbf{D}_\phi {\varvec{\Gamma }}_{\ell } \mathbf{D}_\phi )$ and $\mathbf{G}_\psi = (\mathbf{L}_\psi ^T \mathbf{L}_\psi + \mathbf{D}_\psi {\varvec{\Gamma }}_{\ell } \mathbf{D}_\psi )$, the inversion routine can be written as follows:

1. Set the initial guess $\mathbf{x}_0 = (0,0,\ldots ,0)$.
2. Find a coarse resolution estimate through
  10 $$\begin{aligned} \mathbf{x }^{(\phi )}_{\ell +1} = \mathbf{G}_\phi ^{-1} \mathbf{L}_\phi ^T \mathbf{y} \end{aligned}$$
  with $${\varvec{\Gamma }}_{\ell } = \hbox {diag} ( |\mathbf{D}_\phi \mathbf{x}^{(\phi )} |)^{-1}$$.
3. Find a correction vector belonging to the finer resolution level as
  11 $$\begin{aligned} \mathbf{x }^{(\psi )}_{\ell +1} = \mathbf{G}_\psi ^{-1} \mathbf{L}_\psi ^T ( \mathbf{y} - \mathbf{L}_\phi \mathbf{x}_{\ell +1}^{(\phi )} ) \end{aligned}$$
  with $$ {\varvec{\Gamma }}_{\ell } = \hbox {diag} ( |\mathbf{D}_{\psi } \mathbf{x}^{(\psi )}_{\ell } |)^{-1}$$.
4. Set $$\mathbf{x}_{\ell +1} = \mathbf{x}^{(\phi )}_{\ell +1} + \mathbf{x}^{(\psi )}_{\ell +1}$$ and $$\ell \rightarrow \ell + 1$$.
5. If *n* is smaller than the desired number of iterations, then repeat the steps **(2)**–**(5)**.

If more than two nested meshes are used, then the correction step **(2)** can go through multiple resolution levels $\psi _1, \psi _2, \ldots , \psi _{n_f}$ via the recursive process:

12 $$\begin{aligned} \mathbf{x }^{(\psi _i)}_{\ell +1}\,=\, & {} \mathbf{G}_{\psi _i}^{-1} \mathbf{L}_{\psi _i}^T ( \mathbf{y} - \mathbf{L}_c \mathbf{x}_{\ell +1}^{(c)} \nonumber \\&- \sum _{k = 1}^{i-1} \mathbf{L}_{\psi _i} \mathbf{x}_{\ell +1}^{(\psi _i)}) \end{aligned}$$

with $$ {\varvec{\Gamma }}_{\ell } = \hbox {diag} ( |\mathbf{D}_{\psi _i} \mathbf{x}^{(\psi _i)}_\ell |)^{-1}$$ for $$i = 1, 2, \ldots , n_f$$.

## Abbreviations

1D, 2D, 3D: one, two, three dimensional
ASD-POCS: adaptive-steepest-descent-projection-onto-convex-sets
CBCT: cone beam computed tomography
CG: conjugate gradient
CT: computed tomography
DT-CWT: dual-tree complex wavelet transform
FBP: filtered back-projection
MAR: metal artifact reduction
MRTV-CG: multiresolution conjugate gradient with total variation penalty
MRTV-F: multiresolution with wavelet filtering and total variation penalty
MRTV-H: multiresolution with high total variation penalty
MSE: mean squared error
NaN: not-a-number
RMSE: root mean squared error
ROI: region of interest
PSNR: peak signal-to-noise ratio
SRTV: single resolution with total variation penalty
SRTV-H: single resolution with high total variation penalty
SSIM: structural similarity index
TV: total variation

## Footnotes

1 https://se.mathworks.com/matlabcentral/fileexchange/4551-inpaint-nans.

2 http://www.imp.uni-erlangen.de/forbild/english/results/index.htm.

3 https://physics.nist.gov/PhysRefData/XrayMassCoef/tab4.html.

4 https://physics.nist.gov/PhysRefData/Xcom/html/xcom1.html.

5 https://github.com/CERN/TIGRE.
